# Pretreatment glycemic control status is an independent prognostic factor for cervical cancer patients receiving neoadjuvant chemotherapy for locally advanced disease

**DOI:** 10.1186/s12885-017-3510-3

**Published:** 2017-08-03

**Authors:** Jing Li, Ni-ya Ning, Qun-xian Rao, Rong Chen, Li-juan Wang, Zhong-qiu Lin

**Affiliations:** 1 Department of Gynecologic Oncology, Sun Yat-sen Memorial Hospital, Sun Yat-sen University, 102 Western Yanjiang Road, Guangzhou, 510120 People’s Republic of China; 2 Key Laboratory of Malignant Tumor Gene Regulation and Target Therapy of Guangdong Higher Education Institutes, Sun Yat-sen University, Guangzhou, 510120 People’s Republic of China; 3 Department of Obstetrics and Gynecology, People’s Hospital of Shaolin District, Luohe, 462300 People’s Republic of China; 4 Health center, Sun Yat-sen Memorial Hospital, Sun Yat-sen University, Guangzhou, 510120 People’s Republic of China

**Keywords:** Diabetes mellitus, Hemoglobin A_1c_, Cervical cancer, Neoadjuvant chemotherapy, Prognosis

## Abstract

**Background:**

To investigate whether poor glycemic control status has a negative impact on survival outcomes and tumor response to chemotherapy in patients receiving neoadjuvant chemotherapy (NACT) for locally advanced cervical cancer (LACC).

**Methods:**

A retrospective cohort study was conducted to examine LACC patients undergoing NACT and radical hysterectomy between 2002 and 2011. Patients were divided into three groups: patients without diabetes mellitus (DM), diabetic patients with good glycemic control, and diabetic patients with poor glycemic control. Hemoglobin A_1c_ (HbA_1c_) levels were used to indicate glycemic control status. Recurrence-free survival (RFS), cancer-specific survival (CSS) and overall survival (OS) were analyzed using log-rank tests and Cox proportional hazards models.

**Results:**

In total, 388 patients were included and had a median follow-up time of 39 months (range: 4–67 months). Diabetes mellitus (DM) was diagnosed in 89 (22.9%) patients, only 35 (39.3%) of whom had good glycemic control prior to NACT (HbA_1c_ < 7.0%). In survival analysis, compared with patients with good glycemic control and patients without DM, patients with poor glycemic control (HbA_1c_ ≥ 7.0%) exhibited decreased recurrence-free survival (RFS), cancer-specific survival (CSS) and overall survival (OS). In multivariate analysis, HbA_1c_ ≥ 7.0% was identified as an independent predictor for decreased RFS (hazard ratio [HR] = 3.33, *P* < 0.0001), CSS (HR = 3.60, *P* < 0.0001) and OS (HR = 4.35, *P* < 0.0001). In the subgroup of diabetic patients, HbA_1c_ ≥ 7.0% prior to NACT had an independent negative effect on RFS (HR = 2.18, *P* = 0.044) and OS (HR = 2.29, *P* = 0.012). When examined as a continuous variable, the HbA_1c_ level was independently associated with decreased RFS (HR = 1.39, *P* = 0.002), CSS (HR = 1.28, *P* = 0.021) and OS (HR = 1.27, *P* = 0.004). Both good (odds ratio [OR] = 0.06, *P* < 0.0001) and poor glycemic control (OR = 0.04, *P* < 0.0001) were independently associated with a decreased likelihood of complete response following NACT.

**Conclusions:**

Poor glycemic control is an independent predictor of survival and tumor response to chemotherapy for patients receiving NACT for LACC.

**Electronic supplementary material:**

The online version of this article (doi:10.1186/s12885-017-3510-3) contains supplementary material, which is available to authorized users.

## Background

The Global Cancer Report in 2014 indicates that approximately half of all new cancer cases occur in Asia, mostly in China, and that China’s new cancer cases ranked at the top of the list [1]. In fact, the incidence of cancer has been increasing for decades, and cancer is the leading cause of death in China [2]. Tremendous changes in the lifestyle and environment associated with economic development are important contributors to the increased cancer incidence [3]. In addition, these changes have resulted in a sharp increase in the prevalence of diabetes. China has been the country with the largest burden of diabetes worldwide since 2014 [4]. As a common comorbid medical condition, diabetes mellitus (DM) affects 8–18% of all cancer patients [5]. Previously published data suggest that diabetic patients have worse oncologic outcomes than non-diabetics do [6–13]. Therefore, appropriate diabetic control may have a potential influence for cancer patients.

Cervical cancer is the most common gynecologic malignancy in China [14]. Due to the absence of screening programs, most new cases in China are diagnosed at advanced stages [3]. For cervical cancer patients with locally advanced disease (International Federation of Gynecology and Obstetrics [FIGO] stage IB2 and IIA2), neoadjuvant chemotherapy (NACT) plus radical hysterectomy has been advanced as an effective treatment [15]. A meta-analysis that included 21 randomized trials reported that compared with patients receiving radiotherapy alone patients treated by NACT followed by surgery gain greater survival benefit [16]. Moreover, NACT offers several potential benefits, including eliminating micrometastatic dissemination of the disease and reducing the positivity of lymph nodes, thereby minimizing the need for adjuvant radiotherapy [17–19]. Additionally, for patients with locally advanced cervical cancer (LACC), a complete response (CR) after NACT is independently associated with an improved prognosis [16]. Due to the significant prognostic value, tumor response to NACT has been suggested as a surrogate end-point, which can accurately predict long-term survival outcomes for LACC patients [20].

There are reports that cancer patients with hyperglycemia have a poor response to chemotherapy [6, 21–23]. Moreover, for diabetic cancer patients, poor glycemic control has been observed to negatively influence patient prognosis [7–9, 11, 24]. Among LACC patients, our previous study revealed that hyperglycemia before NACT is an independent predictor of increased risk of relapse and mortality [6]. Despite the evidence, two important questions remain unanswered. Dose adequate blood glucose management offer a survival benefit for LACC patients? Dose glycemic control status impact the response to chemotherapy for LACC patients receiving NACT? Accordingly, we conducted this study to explore whether the glycemic control status influenced oncologic outcomes among LACC patients who underwent NACT and radical hysterectomy.

## Methods

### Settings and study population

After Institutional Review Board approval was obtained from both institutions, a search of clinical databases at Sun Yat-sen Memorial Hospital and the People’s Hospital of Shaolin District was performed. All patients with FIGO stage IB2 and IIA2 cervical cancer (histologically confirmed squamous cell carcinoma, adenocarcinoma and adenosquamous carcinoma) who underwent NACT and type III radical hysterectomy from January 1, 2002 to June 30, 2011 were retrospectively reviewed. Pretreatment informed consent was required for all included patients. Patients younger than 16 years as well as patients who had undergone treatment at other hospitals or who had been treated with chemotherapy or radiation therapy for other malignancies or who did not complete the planned cycles of NACT were excluded from the present analysis.

Two to three cycles of NACT were prescribed. Patients underwent type III radical hysterectomy and pelvic lymphadenectomy within 4 weeks after the administration of the last cycle of NACT. CR was defied as no evidence of viable tumor cells in the tumorous area [25]. Post-surgical adjuvant radiotherapy was prescribed according to Sedlis criteria [15]. DM was defined according to the American Diabetes Association diagnostic criteria or a patient-reported history of diabetes [26]. For comparison, patients were classified into three groups: group I (patients without DM), group II (diabetic patients with good glycemic control, hemoglobin A_1c_ [HbA_1c_] levels prior to NACT <7.0%) and group III (diabetic patients with poor glycemic control, HbA_1c_ levels prior to NACT ≥7.0%) [10, 27]. If there were multiple HbA_1c_ measurements within 6 months before NACT, the mean value was selected for analysis.

The follow-up was scheduled every 3 months for the first 2 years, then every 6 months for the next 3 years, and every year thereafter. Follow-up visits included complete history, physical examination and Papanicolau smear of the vaginal vault. Follow-up information was obtained from office visits or telephone interviews (Additional file 1: Figure S1. Guide for follow-up and telephone interview). When recurrence was suspected based on clinical findings, imaging studies or biopsies of the suspicious lesions were performed on a case by-case basis.

### Statistical analysis

The primary objective of this study was to determine the association between glycemic control status and recurrence-free survival (RFS). The secondary objective was to investigate the impact of glycemic control on cancer-specific survival (CSS), overall survival (OS) and the rate of CR. RFS, CSS and OS were calculated from the date of NACT until the date of events (recurrence OR death from cervical cancer OR death from any cause) or the date of last follow-up. The Kaplan-Meier method was used to estimate the RFS, CSS and OS curves. The log-rank test was used to test for differences between curve estimates. For multiple comparisons of survival curves among the three groups, the Bonferroni adjustment was applied. Cox proportional hazards model using with enter method was used to determine independent prognostic factors. Hazard ratios (HRs) and 95% confidence intervals (CIs) were estimated. The proportional hazards assumption of each variable was verified by Schoenfeld residual plots and no departures from proportionality were observed. Multivariate logistic regression with enter method was used to identify independent variables predicting CR following NACT. Correlations between CR and assessed variables were expressed as odds ratios (ORs) with 95% CI. Variables reaching statistical significance at the *P* < 0.15 level in the univariate analysis were entered into the multivariate model. All statistical tests were two-sided, and a two-tailed *P* value <0.05 was considered statistically significant. Statistical analyses were performed using software (STATA 10.0, special edition; StataCorp, College Station, TX, USA).

## Results

### Patient characteristics

A total of 388 patients met study criteria. Table 1 summarizes the patient demographics. The median patient age was 52 years (range: 24–80), and the median body mass index (BMI) was 23.2 kg/m^2^ (range: 19.4–30.1). Eighty-nine (22.9%) patients had DM. Of these diabetic patients, only 35 (39.3%) had good glycemic control. Group II had three patients with type I DM (8.6%), while group III had six patients with type I DM (11.1%) (*P* = 0.698). At baseline, compared with group I, group II and group III had more patients with higher levels of serum creatinine, lymph node metastasis, parametrial involvement, positive surgical margins, lymphatic vascular space invasion (LVSI), deep stromal invasion, hypertension and cardiovascular diseases. In contrast, group II and group III had fewer patients achieving CR following NACT. Among patients with DM, the median HbA_1c_ level was 7.2% (range: 5.1–12.3%). The median HbA_1c_ level for patients in group II was 6.4% (range: 5.1–6.9%) compared with 8.3% (7.0–12.3%) for patients in group III (*P* = 0.0001). In addition, compared with patients in group II, group III had more patients with a higher level of BMI and serum creatinine, non-squamous cell carcinoma, FIGO IIA2 disease, poor differentiated tumor, parametrial involvement, positive surgical margin, LVIS, deep stromal invasion, cardiovascular diseases and history of using metformin use. More patients in group II achieved CR after NACT compared with group III.

**Table 1:** Patient demographics

	Non-diabetic patients (*n* = 299)	Diabetic patients (*n* = 89)	
		Good Glycemic Control (*n* = 35)	Poor Glycemic Control (*n* = 54)
Age, median (range) (years)	52 (24–80)	52 (28–72)	52 (26–66)
Body mass index (kg/m^2^)	23.2 (20.0–28.5)	23.2 (19.4–26.7)	23.6 (20.8–30.1)
Serum creatinine, median (range) (μmol/l)	69 (43–100)	71 (52–103)	72 (44–121)
Smoking status, n (%)			
Never	278 (93.0)	33 (94.3)	51 (94.4)
Former	9 (3.01)	0 (0.0)	1 (1.9)
Current	1 (0.3)	0 (0.0)	1 (1.9)
Missing data	11 (3.7)	2 (5.7)	1 (1.9)
Regular cervical cancer screening, n (%)			
No	264 (88.3)	29 (82.9)	45 (83.3)
Yes	14 (4.7)	4 (11.4)	7 (13.0)
Missing data	21 (7.0)	2 (5.7)	2 (3.7)
Cell type, n (%)			
Squamous cell carcinoma	253 (84.6)	30 (85.7)	41 (75.9)
Non-squamous cell carcinoma	46.0 (15.4)	5.0 (14.3)	13.0 (24.1)
FIGO stage, n (%)			
IB2	253 (84.6)	30 (85.7)	41 (75.9)
IIA2	46 (15.4)	5 (14.3)	13 (24.1)
Grade, n (%)			
G1–2	274 (91.6)	33 (94.3)	49 (90.7)
G3	25 (8.4)	2 (5.7)	5 (9.3)
Lymph node status, n (%)			
Negative	202 (67.6)	17 (48.6)	28 (51.9)
Positive	97 (32.4)	18 (51.4)	26 (48.2)
Parametrial status, n (%)			
Negative	291 (97.3)	33 (94.3)	50 (92.6)
Positive	8 (2.7)	2 (5.7)	4 (7.4)
Resection margin, n (%)			
Negative	292 (97.7)	32 (91.4)	48 (88.9)
Positive	7 (2.3)	3 (8.6)	6 (11.1)
LVSI, n (%)			
Negative	156 (52.2)	18 (51.4)	26 (48.2)
Positive	143 (47.8)	17 (48.6)	28 (51.9)
Deep stromal invasion, n (%)			
No	97 (32.4)	8 (22.9)	11 (20.4)
Yes	202 (67.6)	27 (77.1)	43 (79.6)
Hypertension, n (%)			
No	241 (80.6)	19 (54.3)	38 (70.4)
Yes	58 (19.4)	16 (45.7)	16 (29.6)
Cardiovascular disease, n (%)			
No	281 (94.0)	30 (85.7)	46 (85.2)
Yes	18 (6.0)	5 (14.3)	8 (14.8)
Metformin use, n (%)			
No	262 (87.6)	31 (88.6)	37 (68.5)
Yes	37 (12.4)	4 (11.4)	17 (31.5)
Complete response, n (%)			
No	96 (32.1)	12 (34.3)	21 (38.9)
Yes	203 (67.9)	23 (65.7)	33 (61.1)

*Abbreviation*: *FIGO* International Federation of Gynecology and Obstetrics, *LVSI* lymphatic vascular space invasion

### Survival outcomes

**Fig. 1:**
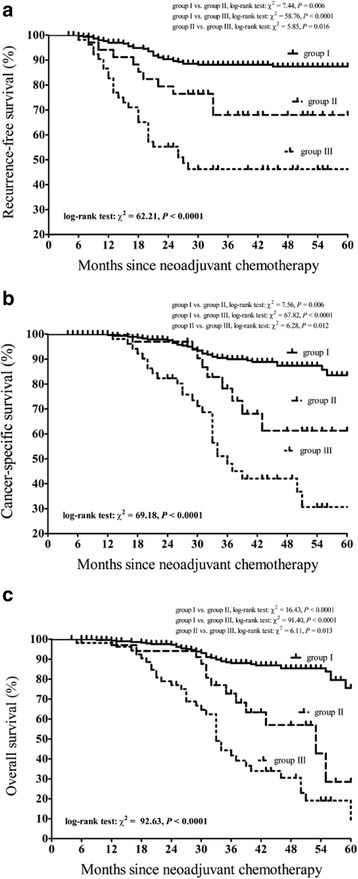
Kaplan-Meier survival curves for survival of cervical cancer patients treated with neoadjuvant chemotherapy and radical hysterectomy for locally advanced disease. **a**. Recurrence-free survival. **b**. Cancer-specific survival. **c**. Overall survival. Patients were stratified by levels of hemoglobin A_1c_ (HbA_1c_). The *P* values were determined by the log-rank test. Group I, patients without diabetes mellitus; group II, patients with well-controlled DM (preoperative HbA_1c_ < 7.0%); group III, patients with poorly controlled DM (preoperative HbA_1c_ ≥ 7.0%)

After a median follow-up time of 39 months (range: 4–67 months), recurrence was observed in 72 patients, including 35 (11.7%) patients in group I, 10 (28.6%) patients in group II and 27 (50%) patients in group III. The differences in recurrence rate between the three groups were significant (*P* < 0.0001). The median time to tumor recurrence was 18 months (range: 6–45 months). RFS at 5 years was 87.3%, 67.9% and 45.2% for patients in group I, group II and group III, respectively. Fig. 1a shows the Kaplan-Meier curves for RFS by diabetic status. Differences between the three survival curves were significant (log-rank test: χ^2^ = 62.21, *P* < 0.0001). A post hoc Bonferroni analysis revealed that the differences in RFS between group I and group II (log-rank test: χ^2^ = 7.44, *P* = 0.006), group I and group III (log-rank test: χ^2^ = 58.76, *P* < 0.0001), and group II and group III (log-rank test: χ^2^ = 5.85, *P* = 0.016) were statistically significant. The cumulative CSS at 5 years was 83.5%, 61.5% and 30.3% for patients in group I, group II and group III, respectively. Fig. 1b displays Kaplan-Meier curves for CSS and significant differences were noted between the curves (log-rank test: χ^2^ = 69.18, *P* < 0.0001). A post hoc Bonferroni analysis showed that the differences in RFS between group I and group II (log-rank test: χ^2^ = 7.56, *P* = 0.006), group I and group III (log-rank test: χ^2^ = 67.82, *P* < 0.0001), and group II and group III (log-rank test: χ^2^ = 6.28, *P* = 0.012) were statistically significant. The estimated 5-year OS was 60.5%, 28.5% and 9.5% for patients in group I, group II and group III, respectively. Kaplan-Meier curves for OS are presented in Fig. 1c, and significant differences were noted between the curves (log-rank test: χ^2^ = 92.63, *P* < 0.0001). In the post hoc Bonferroni analysis, differences in OS between group I and group II (log-rank test: χ^2^ = 16.43, *P* < 0.0001), group I and group III (log-rank test: χ^2^ = 91.40, *P* < 0.0001), and group II and group III (log-rank test: χ^2^ = 6.11, *P* = 0.013) were significant.

The results of Cox regression analyses are detailed in Table 2 and Table 3. HbA_1c_ ≥ 7.0% prior to NACT was identified as an independent predictor of RFS (HR = 3.33, 95% CI 1.89–5.88, *P* < 0.0001), CSS (HR = 3.60, 95% CI 1.96–6.61, *P* < 0.0001) and OS (HR = 4.35, 95% CI 2.64–7.17, *P* < 0.0001).

**Table 2:** Univariate Cox analysis of prognostic factors associated with survival for patients with locally advanced cervical cancer who underwent neoadjuvant chemotherapy and radical hysterectomy

	Recurrence-free survival			Cancer-specific survival			Overall survival		
	HR	95% CI	*P* value	HR	95% CI	*P* value	HR	95% CI	*P* value
Age (years)	1.01	0.98–1.04	0.505	1.01	0.98–1.04	0.390	1.01	0.98–1.03	0.642
Body mass index (kg/m^2^)	1.14	0.99–1.31	0.064	1.12	0.98–1.29	0.105	1.05	0.92–1.19	0.470
Serum creatinine (μmol/l)	1.00	0.98–1.02	0.810	1.01	0.99–1.03	0.618	1.00	0.99–1.02	0.737
Tumor stage (IIA2 vs. IB2)	1.15	0.73–1.83	0.541	1.15	0.71–1.84	0.576	0.97	0.64–1.47	0.873
Histology (non-squamous vs. squamous)	1.69	0.98–2.91	0.059	1.84	1.06–3.19	0.029	1.91	1.19–3.08	0.008
Tumor differentiation (G3 vs. G1–2)	1.49	0.72–3.11	0.285	1.81	0.90–3.65	0.098	1.50	0.78–2.90	0.228
Deep stromal invasion (yes vs. no)	1.95	1.09–3.50	0.025	2.09	1.16–3.77	0.014	2.19	1.30–3.68	0.003
LVSI (yes vs. no)	2.10	1.29–3.41	0.003	2.04	1.25–3.35	0.005	1.62	1.06–2.48	0.025
Positive margins (yes vs. no)	9.37	5.19–16.91	<0.0001	10.2	5.59–18.63	<0.0001	7.92	4.41–14.22	<0.0001
Positive nodes (yes vs. no)	7.13	4.09–12.44	<0.0001	7.55	4.25–13.42	<0.0001	4.81	3.07–7.54	<0.0001
Positive parametrium (yes vs. no)	10.88	5.68–20.87	<0.0001	11.83	6.11–22.87	<0.0001	9.19	4.81–17.55	<0.0001
Diabetes									
No	Reference	Reference	Reference	Reference	Reference	Reference	Reference	Reference	Reference
HbA_1c_ < 7%	2.66	1.32–5.37	0.006	2.79	1.31–5.97	0.008	3.33	1.78–6.24	<0.0001
HbA_1c_ ≥ 7%	6.02	3.63–9.96	<0.0001	7.13	4.17–12.17	<0.0001	7.01	4.44–11.06	<0.0001
Hypertension (yes vs. no)	1.37	0.82–2.27	0.227	1.36	0.78–2.37	0.273	1.31	0.83–2.09	0.248
Cardiovascular disease (yes vs. no)	1.09	0.47–2.52	0.837	0.47	0.18–1.19	0.109	1.11	0.54–2.31	0.770
Metformin (yes vs. no)	1.21	0.65–2.24	0.552	0.88	0.46–1.68	0.705	1.86	1.13–3.07	0.015
Complete response (yes vs. no)	0.52	0.33–0.82	0.005	0.50	0.31–0.83	0.007	0.69	0.45–1.06	0.089

*Abbreviation*: *CI* confidence interval, *HbA*_*1c*_ hemoglobin A_1c_, *HR* hazard ratio, *LVSI* lymphatic vascular space invasion

**Table 3:** Multivariate Cox analysis of prognostic factors associated with survival for patients with locally advanced cervical cancer who underwent neoadjuvant chemotherapy and radical hysterectomy

	Recurrence-free survival			Cancer-specific survival			Overall survival		
	HR	95% CI	*P* value	HR	95% CI	*P* value	HR	95% CI	*P* value
Age (years)									
Body mass index (kg/m^2^)	1.04	0.90–1.19	0.621	1.03	0.89–1.20	0.680			
Serum creatinine (μmol/l)									
Tumor stage (IIA2 vs. IB2)									
Histology (non-squamous vs. squamous)	1.28	0.70–2.35	0.427	1.30	0.68–2.47	0.430	1.28	0.51–3.21	0.602
Tumor differentiation (G3 vs. G1–2)				4.16	1.93–8.97	<0.0001			
Deep stromal invasion (yes vs. no)	0.77	0.40–1.49	0.433	0.83	0.42–1.63	0.586	1.21	0.68–2.16	0.516
LVSI (yes vs. no)	1.26	0.73–2.15	0.405	1.16	0.66–2.04	0.616	0.91	0.56–1.48	0.708
Positive margins (yes vs. no)	3.70	1.86–7.35	<0.0001	4.42	2.15–9.08	<0.0001	3.26	1.69–6.28	<0.0001
Positive nodes (yes vs. no)	5.21	2.82–9.62	<0.0001	6.67	3.47–12.80	<0.0001	3.47	2.09–5.78	<0.0001
Positive parametrium (yes vs. no)	3.37	1.67–6.82	0.001	3.14	1.54–6.41	0.002	2.84	1.41–5.73	0.004
Diabetes									
No	Reference	Reference	Reference	Reference	Reference	Reference	Reference	Reference	Reference
HbA_1c_ < 7%	1.49	0.69–3.22	0.307	1.57	0.68–3.62	0.290	2.06	1.04–4.07	0.039
HbA_1c_ ≥ 7%	3.33	1.89–5.88	<0.0001	3.60	1.96–6.61	<0.0001	4.35	2.64–7.17	<0.0001
Hypertension (yes vs. no)									
Cardiovascular disease (yes vs. no)				0.57	0.22–1.48	0.245			
Metformin (yes vs. no)							1.13	0.41–3.11	0.807
Complete response (yes vs. no)	0.58	0.36–0.95	0.031	0.52	0.31–0.87	0.013	0.78	0.50–1.22	0.279

*Abbreviation*: *CI* confidence interval, *HbA*_*1c*_ hemoglobin A_1c_, *HR* hazard ratio, *LVSI* lymphatic vascular space invasion

To investigate the survival impact of glycemic control for diabetic patients, we excluded patients without DM and performed a further subgroup analyses. The results of Cox proportional hazard analyses are summarized in Table 4 and Table 5. When the level of HbA_1c_ was treated as a dichotomous variable, HbA_1c_ ≥ 7.0% prior to NACT exhibited an independent negative effect on RFS (HR = 2.18, 95% CI 1.02–4.63, *P* = 0.044) and OS (HR = 2.29, 95% CI 1.20–4.35, *P* = 0.012). When the HbA_1c_ level was examined as a continuous variable (Additional file 2: Table S1), it was independently associated with RFS (HR = 1.39, 95% CI 1.13–1.71, *P* = 0.002), CSS (HR = 1.28, 95% CI 1.04–1.59, *P* = 0.021) and OS (HR = 1.27, 95% CI 1.08–1.50, *P* = 0.004).

**Table 4:** Univariate Cox analysis of prognostic factors associated with survival for diabetic patients with locally advanced cervical cancer who underwent neoadjuvant chemotherapy and radical hysterectomy

	Univariate analysis			Univariate analysis			Univariate analysis		
	HR	95% CI	*P* value	HR	95% CI	*P* value	HR	95% CI	*P* value
Age (years)	1.00	0.96–1.04	0.875	1.01	0.97–1.05	0.681	0.99	0.96–1.03	0.698
Body mass index (kg/m^2^)	1.17	0.99–1.39	0.065	1.18	1.00–1.39	0.055	1.08	0.92–1.26	0.361
Serum creatinine (μmol/l)	1.00	0.98–1.02	0.872	1.02	0.99–1.04	0.211	1.01	0.98–1.03	0.610
Tumor stage (IIA2 vs. IB2)	2.04	1.07–3.90	0.031	1.85	0.96–3.57	0.065	1.44	0.82–2.54	0.207
Histology (non-squamous vs. squamous)	1.31	0.62–2.77	0.485	1.27	0.58–2.80	0.549	1.40	0.71–2.76	0.332
Tumor differentiation (G3 vs. G1–2)	0.79	0.19–3.30	0.751	0.90	0.22–3.76	0.888	1.07	0.33–3.44	0.913
Deep stromal invasion (yes vs. no)	1.49	0.62–3.58	0.371	1.55	0.68–3.55	0.300	1.79	0.86–3.70	0.119
LVSI (yes vs. no)	1.16	0.61–2.24	0.647	1.26	0.65–2.44	0.503	0.96	0.54–1.70	0.890
Positive margins (yes vs. no)	3.69	1.71–7.97	0.001	4.14	1.88–9.13	<0.0001	3.04	1.42–6.47	0.004
Positive nodes (yes vs. no)	4.81	2.19–10.56	<0.0001	4.12	1.87–9.07	<0.0001	3.07	1.63–5.78	<0.0001
Positive parametrium (yes vs. no)	5.55	2.27–13.55	<0.0001	4.76	1.95–11.59	0.001	3.71	1.55–8.89	0.003
Diabetic status (HbA_1c_ ≥ 7% vs. HbA_1c_ < 7%)	2.24	1.08–4.63	0.030	2.45	1.15–5.22	0.020	2.17	1.15–4.11	0.017
Hypertension (yes vs. no)	0.90	0.46–1.77	0.763	0.94	0.47–1.86	0.854	0.90	0.50–1.62	0.723
Cardiovascular disease (yes vs. no)	0.61	0.22–1.73	0.355	0.51	0.18–1.46	0.211	0.68	0.30–1.52	0.343
Metformin (yes vs. no)	0.77	0.34–1.75	0.531	0.79	0.33–1.90	0.595	1.22	0.62–2.40	0.563
Complete response (yes vs. no)	0.56	0.29–1.07	0.077	0.49	0.25–0.94	0.033	0.74	0.41–1.33	0.316

*Abbreviation*: *CI*, confidence interval, *HbA*_*1c*_ hemoglobin A_1c_, *HR* hazard ratio, *LVSI* lymphatic vascular space invasion

**Table 5:** Multivariate Cox analysis of prognostic factors associated with survival for diabetic patients with locally advanced cervical cancer who underwent neoadjuvant chemotherapy and radical hysterectomy

	Recurrence-free survival			Cancer-specific survival			Overall survival		
	HR	95% CI	*P* value	HR	95% CI	*P* value	HR	95% CI	*P* value
Age (years)									
Body mass index (kg/m^2^)	1.16	0.94–1.44	0.171	1.12	0.90–1.38	0.312			
Serum creatinine (μmol/l)									
Tumor stage (IIA2 vs. IB2)	1.64	0.78–3.48	0.195	1.55	0.71–3.37	0.267			
Histology (non-squamous vs. squamous)									
Tumor differentiation (G3 vs. G1–2)									
Deep stromal invasion (yes vs. no)							1.16	0.54–2.52	0.702
LVSI (yes vs. no)									
Positive margins (yes vs. no)	2.25	0.94–5.39	0.070	3.21	1.31–7.90	0.011	2.16	1.00–4.68	0.050
Positive nodes (yes vs. no)	3.64	1.49–8.85	0.004	3.44	1.42–8.35	0.006	2.73	1.37–5.45	0.004
Positive parametrium (yes vs. no)	3.50	1.31–9.32	0.012	2.71	1.00–7.35	0.049	2.16	0.88–5.30	0.093
Diabetic status (HbA_1c_ ≥ 7% vs. HbA_1c_ < 7%)	2.18	1.02–4.63	0.044	2.00	0.89–4.49	0.092	2.29	1.20–4.35	0.012
Hypertension (yes vs. no)									
Cardiovascular disease (yes vs. no)									
Metformin (yes vs. no)									
Complete response (yes vs. no)	0.82	0.39–1.72	0.599	0.64	0.30–1.36	0.247			

*Abbreviation*: *CI*, confidence interval, *HbA*_*1c*_ hemoglobin A_1c_, *HR* hazard ratio, *LVSI* lymphatic vascular space invasion

### Factors predicting CR after NACT

CR following NACT has been validated as an accurate surrogate endpoint of survival for LACC patients. Given its significance, a further logistic regression analysis was conducted to identify variables that could predict CR after NACT. Table 6 presents the results. Both good glycemic control (OR = 0.06, 95% CI 0.02–0.17, *P* < 0.0001) and poor glycemic control (OR = 0.04, 95% CI 0.02–0.11, *P* < 0.0001) were identified as independent predictors of decreasing incidence of CR after NACT.

**Table 6:** Univariate and multivariate analysis of predictors of complete response following neoadjuvant chemotherapy in patients with locally advanced cervical cancer who underwent neoadjuvant chemotherapy and radical hysterectomy

	Complete response					
	Univariate analysis			Multivariate analysis		
	OR	95% CI	*P* value	OR	95% CI	*P* value
Age (years)	1.01	0.99–1.04	0.286			
Body mass index (kg/m^2^)	1.19	1.01–1.39	0.037	1.27	1.04–1.55	0.020
Serum creatinine (μmol/l)	0.97	0.95–0.99	0.001	0.99	0.97–1.02	0.573
Tumor stage (IIA2 vs. IB2)	1.60	1.12–2.30	0.010	0.99	0.73–1.35	0.956
Histology (non-squamous vs. squamous)	1.55	0.64–3.71	0.329			
Tumor differentiation (G3 vs. G1–2)	0.91	0.45–1.84	0.784			
Diabetes						
No	Reference	Reference	Reference	Reference	Reference	Reference
HbA_1c_ < 7%	0.05	0.02–0.14	<0.0001	0.06	0.02–0.17	<0.0001
HbA_1c_ ≥ 7%	0.04	0.02–0.10	<0.0001	0.04	0.02–0.11	<0.0001
Hypertension (yes vs. no)	0.34	0.20–0.59	<0.0001	0.38	0.20–0.74	0.004
Cardiovascular disease (yes vs. no)	0.84	0.35–2.04	0.700			
Metformin (yes vs. no)	0.64	0.34–1.22	0.174			

*Abbreviation*: *CI* confidence interval, *HbA*_*1c*_ hemoglobin A_1c_, *OR* odds ratio

## Discussion

The prevalence of DM is 9.7% in mainland China, which translates into 92.4 million adults with diabetes [28]. Of patients included in the present study, 89 (22.9%) had DM, and only 35 (39.3%) diabetic patients had good glycemic control. Our results were consistent with previous reports [5]. Considering the negative impact of DM on the prognosis for cancer patients, we conducted this study. We found that poor glycemic control (HbA_1c_ ≥ 7.0%) was independently associated with an increased risk of tumor recurrence and death in LACC patients who received NACT. In addition, DM patients, regardless of the glycemic control status, were less likely to achieve CR after NACT than were non-diabetics.

Previous studies assessed the influence of glycemic control status on survival outcomes in cancer patients. In diabetic patients receiving curative resection for hepatitis C virus-related hepatocellular carcinoma, poor glycemic control was an independent predictor of relapse following surgery [8]. In patients with colorectal cancer, poorly controlled DM independently predicted more advanced disease and poor 5-year survival [11]. For patients with early-stage breast cancer, mortality was significantly increased in women with HbA_1C_ ≥ 7.0% compared with women with HbA_1C_ less than 6.5% [10]. For patients with urinary system tumors, poor glycemic control was observed as a prognostic factor of poor prognosis [7, 9, 12, 24]. For cervical cancer patients, previously published data have suggested a deleterious effect of hyperglycemia on patient survival. However, no study to date has evaluated the survival impact of glycemic control status among cervical cancer patients.

The potential influence of glycemic control on cancer prognosis is complex. Because glucose uptake in cancer cells is significantly enhanced, patients with poorly controlled DM exhibit increased tumor cell proliferation [29, 30]. Second, as a result of poor glycemic control, hyperglycemia can induce elevated levels of insulin and insulin-like growth and inflammatory factors, which can directly augment tumor progression [10, 31, 32]. Third, poor glycemic control enhances the production of advanced glycosylated end products. Consequently, lipid peroxidation and the production of genotoxic aldehyde can be induced, which result in DNA damage [11]. Fourth, cancer patients with DM frequently have comorbid conditions, which often lead clinicians to follow less aggressive cancer treatments [33, 34]. Additionally, diabetic women underuse cervical cancer screening as compared with non-diabetic women, which may lead to tumor detection at a later stage on diagnosis [35].

For patients with LACC, the prognostic value of CR following NACT has been validated by previous studies. A meta-analysis by Ye et al. including data from 18 studies reported that response to NACT is an indicator of significantly improved prognosis [16]. Alessandro et al. conducted a retrospective cohort study, which is one of the largest samples of LACC patients with robust follow-up data (median follow-up time: 12.7 years) [20]. They assessed the long-term significance of tumor response to NACT, and reported that CR following NACT could be used as a reliable surrogate end-point, which accurately predicts significantly improved prognosis for LACC patients undergoing NACT. The clinical significance of CR following NACT was also confirmed by our study. Moreover, compared with patients without DM, both DM patients with good glycemic control and DM patients with poor glycemic control were less likely to achieve CR. Our findings were in agreement with previous reports, where a poor response to chemotherapy was more frequently observed in patients with hyperglycemia [6, 21–23].

The present study has several limitations. The most important limitation derives from its retrospective design which may have missed confounding factors. For instance, some demographic and lifestyle variables such as patient income and alcohol habits were not included. In addition, our study did not answer whether the duration of DM and the glycemic control status could impact patient survival. Second, a central pathology review for surgical specimens was not performed. Third, data on HbA_1c_ levels in patients without DM were unavailable. Therefore, some patients in group I might have been true diabetic patients, which could induce selection bias. Fourth, although we assessed the influence of metformin, the effects of other anti-diabetic drugs were not analyzed. However, to the best of our knowledge, the present work is the first to show the impact of glycemic control as represented by HbA_1c_ levels on cancer treatment outcomes in LACC patients receiving NACT and radical hysterectomy. Moreover, our large sample size allowed us to provide reliable evidence to inform clinical practice.

## Conclusions

In summary, our findings suggest that poor glycemic control prior to NACT, as indicated by elevated HbA_1C_ levels, is independently associated with an increased risk of tumor recurrence and mortality for LACC patient. We also present evidence that poorly controlled DM is an independent predictor of poor response to NACT. Given these results, we believe that the proper management of diabetes may present an opportunity for improving survival outcomes for LACC patients. Our findings should be confirmed prospectively. Future randomized trials are also warranted to investigate whether targeting glycemic control offers oncological benefits for diabetic LACC patients.

## Additional files

Additional file 1: Figure S1. Guide for follow-up and telephone interview. (EPS 650 kb)

Additional file 2: Table S1. Univariate and multivariate Cox analysis of prognostic factors associated with survival for diabetic patients with locally advanced cervical cancer who underwent neoadjuvant chemotherapy and radical hysterectomy. (DOCX 23 kb)

## Abbreviations

BMI: Body mass index
CI: Confidence interval
CR: Complete response
CSS: Cancer-specific survival
DM: Diabetes mellitus
FIGO: International Federation of Gynecology and Obstetrics
HbA_1c_: Hemoglobin A_1c_
HR: Hazard ratio
LACC: Locally advanced cervical cancer
LVSI: Lymphatic vascular space invasion
NACT: Neoadjuvant chemotherapy
OR: Odds ratio
OS: Overall survival
RFS: Recurrence-free survival
