# Supplementary material for: Pretreatment glycemic control status is an independent prognostic factor for cervical cancer patients receiving neoadjuvant chemotherapy for locally advanced disease
Source: BMC Cancer. 2017 Aug 3;17:517. doi: 10.1186/s12885-017-3510-3 (PMC5543538; doi:10.1186/s12885-017-3510-3)
Supplement: Supplementary file 2 — Univariate and multivariate Cox analysis of prognostic factors associated with survival for diabetic patients with locally advanced cervical cancer who underwent neoadjuvant chemotherapy and radical hysterectomy. (DOCX 23 kb) [file 12885_2017_3510_MOESM2_ESM.docx]

Supplementary Table 1. Univariate and multivariate Cox analysis of prognostic factors associated with survival for diabetic patients with locally advanced cervical cancer who underwent neoadjuvant chemotherapy and radical hysterectomy

|  | Recurrence-free survival | | | | | | Cancer-specific survival | | | | | | Overall survival | | | | | | |
| --- | --- | --- | --- | --- | --- | --- | --- | --- | --- | --- | --- | --- | --- | --- | --- | --- | --- | --- | --- |
|  | Univariate analysis | | | Multivariate analysis | | | Univariate analysis | | | Multivariate analysis | | | Univariate analysis | | | Multivariate analysis | | | |
|  | HR | 95% CI | *P* value | HR | 95% CI | *P* value | HR | 95% CI | *P* value | HR | 95% CI | *P* value | HR | 95% CI | P value | HR | 95% CI | *P* value |  |
| Age (years) | 1.00 | 0.96-1.04 | 0.875 |  |  |  | 1.01 | 0.97-1.05 | 0.681 |  |  |  | 0.99 | 0.96-1.03 | 0.698 |  |  |  |  |
| Body mass index (kg/m^2^) | 1.17 | 0.99-1.39 | 0.065 | 1.09 | 0.89-1.34 | 0.401 | 1.18 | 1.00-1.39 | 0.055 | 1.05 | 0.85-1.30 | 0.624 | 1.08 | 0.92-1.26 | 0.361 |  |  |  |  |
| Serum creatinine (μmol/l) | 1.00 | 0.98-1.02 | 0.872 |  |  |  | 1.02 | 0.99-1.04 | 0.211 |  |  |  | 1.01 | 0.98-1.03 | 0.610 |  |  |  |  |
| Tumor stage (IIA2 vs. IB2) | 2.04 | 1.07-3.90 | 0.031 | 1.60 | 0.75-3.41 | 0.228 | 1.85 | 0.96-3.57 | 0.065 | 1.49 | 0.68-3.27 | 0.317 | 1.44 | 0.82-2.54 | 0.207 |  |  |  |  |
| Histology (non-squamous vs. squamous) | 1.31 | 0.62-2.77 | 0.485 |  |  |  | 1.27 | 0.58-2.80 | 0.549 |  |  |  | 1.40 | 0.71-2.76 | 0.332 |  |  |  |  |
| Tumor differentiation (G3 vs. G1-2) | 0.79 | 0.19-3.30 | 0.751 |  |  |  | 0.90 | 0.22-3.76 | 0.888 |  |  |  | 1.07 | 0.33-3.44 | 0.913 |  |  |  |  |
| Deep stromal invasion (yes vs. no) | 1.49 | 0.62-3.58 | 0.371 |  |  |  | 1.55 | 0.68-3.55 | 0.300 |  |  |  | 1.79 | 0.86-3.70 | 0.119 | 1.29 | 0.60-2.77 | 0.515 |  |
| LVSI (yes vs. no) | 1.16 | 0.61-2.24 | 0.647 |  |  |  | 1.26 | 0.65-2.44 | 0.503 |  |  |  | 0.96 | 0.54-1.70 | 0.890 |  |  |  |  |
| Positive margins (yes vs. no) | 3.69 | 1.71-7.97 | 0.001 | 2.40 | 0.97-5.97 | 0.059 | 4.14 | 1.88-9.13 | <0.0001 | 3.52 | 1.38-8.95 | 0.008 | 3.04 | 1.42-6.47 | 0.004 | 2.31 | 1.06-5.06 | 0.036 |  |
| Positive nodes (yes vs. no) | 4.81 | 2.19-10.56 | <0.0001 | 3.80 | 1.55-9.31 | 0.003 | 4.12 | 1.87-9.07 | <0.0001 | 3.57 | 1.45-8.79 | 0.006 | 3.07 | 1.63-5.78 | <0.0001 | 2.66 | 1.34-5.28 | 0.005 |  |
| Positive parametrium (yes vs. no) | 5.55 | 2.27-13.55 | <0.0001 | 2.93 | 1.10-7.82 | 0.032 | 4.76 | 1.95-11.59 | 0.001 | 2.45 | 0.91-6.61 | 0.077 | 3.71 | 1.55-8.89 | 0.003 | 1.88 | 0.75-4.70 | 0.178 |  |
| Diabetic status (HbA_1c_ was treated as a continuous variable) | 1.45 | 1.20-1.75 | <0.0001 | 1.39 | 1.13-1.71 | 0.002 | 1.36 | 1.14-1.63 | 0.001 | 1.28 | 1.04-1.59 | 0.021 | 1.26 | 1.07-1.48 | 0.005 | 1.27 | 1.08-1.50 | 0.004 |  |
| Hypertension (yes vs. no) | 0.90 | 0.46-1.77 | 0.763 |  |  |  | 0.94 | 0.47-1.86 | 0.854 |  |  |  | 0.90 | 0.50-1.62 | 0.723 |  |  |  |  |
| Cardiovascular disease (yes vs. no) | 0.61 | 0.22-1.73 | 0.355 |  |  |  | 0.51 | 0.18-1.46 | 0.211 |  |  |  | 0.68 | 0.30-1.52 | 0.343 |  |  |  |  |
| Metformin (yes vs. no) | 0.77 | 0.34-1.75 | 0.531 |  |  |  | 0.79 | 0.33-1.90 | 0.595 |  |  |  | 1.22 | 0.62-2.40 | 0.563 |  |  |  |  |
| Complete response (yes vs. no) | 0.56 | 0.29-1.07 | 0.077 | 0.82 | 0.40-1.68 | 0.589 | 0.49 | 0.25-0.94 | 0.033 | 0.61 | 0.30-1.26 | 0.180 | 0.74 | 0.41-1.33 | 0.316 |  |  |  |  |
